# Medical Misinformation in Polish on the World Wide Web During the COVID-19 Pandemic Period: Infodemiology Study

**DOI:** 10.2196/48130

**Published:** 2024-03-29

**Authors:** Małgorzata Chlabicz, Aleksandra Nabożny, Jolanta Koszelew, Wojciech Łaguna, Anna Szpakowicz, Paweł Sowa, Wojciech Budny, Katarzyna Guziejko, Magdalena Róg-Makal, Sławomir Pancewicz, Maciej Kondrusik, Piotr Czupryna, Beata Cudowska, Dariusz Lebensztejn, Anna Moniuszko-Malinowska, Adam Wierzbicki, Karol A Kamiński

**Affiliations:** 1 Department of Population Medicine and Lifestyle Diseases Prevention Medical University of Białystok Białystok Poland; 2 Department of Invasive Cardiology Medical University of Białystok Białystok Poland; 3 Department of Software Engineering Gdańsk University of Technology Gdańsk Poland; 4 R&D Department Science4People Limited Liability Company Szczecin Poland; 5 Faculty of Computer Science Bialystok University of Technology Białystok Poland; 6 Department of Cardiology Medical University of Bialystok Białystok Poland; 7 Department of Allergology and Internal Medicine Medical University of Białystok Białystok Poland; 8 2nd Department of Lung Diseases and Tuberculosis Medical University of Białystok Białystok Poland; 9 Department of Infectious Diseases and Neuroinfection Medical University of Białystok Białystok Poland; 10 Department of Pediatrics, Gastroenterology, Hepatology, Nutrition, Allergology and Pulmonology Medical University of Bialystok Białystok Poland; 11 Department of Computer Science Polish-Japaneese Academy of Information Technology Warsaw Poland

**Keywords:** infodemic, fake news, information credibility, online health information, evidence based medicine, EBM, false, credibility, credible, health information, online information, information quality, infoveillance, infodemiology, misinformation, disinformation

## Abstract

**Background:**

Although researchers extensively study the rapid generation and spread of misinformation about the novel coronavirus during the pandemic, numerous other health-related topics are contaminating the internet with misinformation that have not received as much attention.

**Objective:**

This study aims to gauge the reach of the most popular medical content on the World Wide Web, extending beyond the confines of the pandemic. We conducted evaluations of subject matter and credibility for the years 2021 and 2022, following the principles of evidence-based medicine with assessments performed by experienced clinicians.

**Methods:**

We used 274 keywords to conduct web page searches through the BuzzSumo Enterprise Application. These keywords were chosen based on medical topics derived from surveys administered to medical practitioners. The search parameters were confined to 2 distinct date ranges: (1) January 1, 2021, to December 31, 2021; (2) January 1, 2022, to December 31, 2022. Our searches were specifically limited to web pages in the Polish language and filtered by the specified date ranges. The analysis encompassed 161 web pages retrieved in 2021 and 105 retrieved in 2022. Each web page underwent scrutiny by a seasoned doctor to assess its credibility, aligning with evidence-based medicine standards. Furthermore, we gathered data on social media engagements associated with the web pages, considering platforms such as Facebook, Pinterest, Reddit, and Twitter.

**Results:**

In 2022, the prevalence of unreliable information related to COVID-19 saw a noteworthy decline compared to 2021. Specifically, the percentage of noncredible web pages discussing COVID-19 and general vaccinations decreased from 57% (43/76) to 24% (6/25) and 42% (10/25) to 30% (3/10), respectively. However, during the same period, there was a considerable uptick in the dissemination of untrustworthy content on social media pertaining to other medical topics. The percentage of noncredible web pages covering cholesterol, statins, and cardiology rose from 11% (3/28) to 26% (9/35) and from 18% (5/28) to 26% (6/23), respectively.

**Conclusions:**

Efforts undertaken during the COVID-19 pandemic to curb the dissemination of misinformation seem to have yielded positive results. Nevertheless, our analysis suggests that these interventions need to be consistently implemented across both established and emerging medical subjects. It appears that as interest in the pandemic waned, other topics gained prominence, essentially “filling the vacuum” and necessitating ongoing measures to address misinformation across a broader spectrum of health-related subjects.

## Introduction

### Background

Although disinformation has been recognized since the early days of the printing press in previous centuries, its significance has grown substantially in the era of the internet. Technology historian Melvin Kranzberg aptly remarked, “Technology is neither inherently good nor bad; nor is it neutral.” [1]. Similarly, the internet, with its free access to information through the World Wide Web (WWW), not only offers numerous advantages but also facilitates the rapid spread of noncredible or fake stories. Disinformation poses a threat to society across various aspects of life, with particular significance in the realm of online health information. The dissemination of false health-related information can significantly impact individuals’ well-being and potentially result in long-term consequences for their health [2]. This study aims to identify medicine-related topics that contribute to the proliferation of unreliable content on the web, such as those related to the COVID-19 pandemic, vaccination, statins, and diet. Additionally, we sought to observe the dynamics of these topics over a 2-year period (2021-2022).

The years 2020, 2021, and 2022 were largely overshadowed by the SARS-CoV-2 pandemic in the realm of medicine-related online health information. Consequently, it is unsurprising that investigations into the dissemination of medical disinformation during this period predominantly centered around topics associated with COVID-19. Furthermore, the research placed a strong emphasis on the perspective of the general public, rather than that of medical practitioners who grappled with the consequences of the widespread dissemination of disinformation. Our study endeavored to address 2 key challenges: first, to comprehend online health information beyond the scope of the pandemic, and second, to investigate noncredible online health information through the lens of medical practitioners.

The inception of our study involved gathering surveys from doctors, outlining the topics they encountered in their clinical practice. Consequently, we obtained information about false content that significantly influenced patients’ attitudes toward medical recommendations. Additionally, we collected data on disinformation concerning the COVID-19 pandemic and 6 other medical topics, presenting a comprehensive overview of the overall dynamics of the spread of medical online disinformation. Our approach involved a systematic search for the most widely read articles and social media engagements pertaining to selected topics in 2021 and 2022. Subsequently, a team of experienced clinicians assessed the credibility of these sources, adhering to the principles of evidence-based medicine. This methodology enabled us to pinpoint crucial areas for enhancing interventions aimed at curbing the dissemination of noncredible online health information.

### Literature Review

Although various definitions of information credibility [3] and noncredible information, including disinformation or misinformation, exist [4], there remains a lack of a clear definition for false information. In some instances, the literature uses the term “medical fake news” [5], which is defined as “news that is intentionally false and could mislead readers” and “information discrepant with medical knowledge” [6]. The primary objective of such misinformation is to manipulate and shape public opinion. Numerous publications highlight the adverse repercussions of disinformation spreading on the internet within communities [7,8]. An example is the study conducted by Betsch et al [9], which revealed that spending just 5-10 minutes on vaccine-critical websites heightened the perception of vaccine risk. This, in turn, led to negative perceptions of vaccination risks and a decreased intention to vaccinate.

Evidence-based medicine furnishes factual information that is not only relevant to everyday medical practice but also invaluable for experts endeavoring to refute medical fake news. Coined in 1991 [10], evidence-based medicine is defined as “the conscientious, explicit, and judicious use of current best evidence in making decisions about the care of individual patients” [11]. Evidence-based medicine aims to integrate the clinician’s experience, the patient’s values, and the best available scientific information to inform clinical management decisions. The principles of evidence-based medicine apply not only to individual medical practice but also to institutions and the health care system at large.

The novel coronavirus, known as SARS-CoV-2, gave rise to COVID-19. Beyond causing the COVID-19 pandemic, it served as a catalyst for extensive medical disinformation campaigns [12-14]. The internet became inundated with millions of posts about COVID-19 [15]. The challenges associated with developing accurate diagnostic tests, therapeutic protocols, and methods for preventing SARS-CoV-2 infections have created fertile ground for manipulation and disinformation, particularly in health decision-making [13-15]. False information about the pandemic proliferated through mass media, social platforms, and text messaging applications, leading to various adverse effects such as (1) reducing patients’ willingness to vaccinate, (2) obstructing measures to contain disease outbreaks, and (3) instigating the physical interruption of access to health care, among others [16].

The proliferation of fake news about COVID-19 has significantly contributed to the dissemination of disinformation in related fields, notably in the context of vaccination, putting scientific knowledge to the test [17,18]. While the antivaccination movement had already propagated conspiracy theories, such as the notion that large pharmaceutical companies and other vested interests exaggerate vaccination benefits while concealing risks or dangers [19], the pandemic has heightened the phenomenon of skepticism toward immunization. Vaccine hesitancy was previously noted in the cases of well-known vaccinations such as the MMR (measles, mumps, and rubella) vaccine [20] and the flu vaccine [21]. Research has indicated that attitudes and conspiracy beliefs, which can be viewed as a particular set of behaviors, are correlated with the willingness to vaccinate. For instance, Dubé et al [22] demonstrated that parents who chose to vaccinate their children were more likely to receive information about vaccines from health care professionals and less inclined to seek information on the internet. Additionally, Sommariva et al [23] found that disinformation circulating on social networks could impede disease prevention efforts. Regarding COVID-19 vaccines, new conspiracy theories have surfaced, such as microchip injection, unchecked adverse effects, absence of safety assessment before vaccine distribution, and unproven vaccine effectiveness. Studies indicate that exposure to fake news diminishes the intention to receive the COVID-19 vaccine [24]. Even brief exposure to false information has been shown to potentially modify or influence human behavior [22]. As a result, conspiracy beliefs, trust in information, and brief exposure to online news can impact individuals’ vaccination choices [24]. Findings from a randomized controlled trial by Loomba et al [25] indicated that exposure to online disinformation about COVID-19 vaccines led to a significant decline in vaccination intent, in the samples from both the United States (6.4% points) and the United Kingdom (6.2% points).

The issue of disinformation in cardiology, particularly regarding statins, has been previously documented [26,27]. Although overshadowed during the COVID-19 pandemic, it is resurfacing. Waszak et al [28] conducted an analysis of topics drawing public attention between 2012 and 2017. The primary subjects were cancer, vaccination, heart attack, stroke, HIV/AIDS, hypertension, and diabetes. Vaccination emerged as the topic most affected by fake news, with a staggering 90%, followed by hypertension and HIV/AIDS, both at 70%.

The literature has delved into the analysis of medical information, particularly those related to nutrition, that has been deemed false or misleading [7,29]. While discussions regarding the impact of disinformation and misinformation on knowledge levels and nutritional decision-making are commonplace, there is a relatively infrequent analysis of the extent of such content on the internet [30]. Several efforts have been made to quantify the phenomenon of nutrition disinformation during the pandemic period. For instance, a Turkish study estimated that approximately 20% of analyzed YouTube videos on nutrition topics contained fake news [31], while a Korean study reported as much as 37% [32]. From a qualitative perspective, nutrition misinformation during the COVID-19 pandemic era encompassed topics related to food immunity boosters, purportedly designed to reduce the risk or severity of infection. Studies have explored the impact of supplementation with zinc, vitamin C, vitamin A, herbs, or nutraceuticals on mitigating the mentioned risks [33,34]. An Indian study observed that over 70% of respondents reflected immunity-boosting food in their dietary decisions [8]. The pandemic led to a surge in the circulation of fake news concerning the supposed protective effects of alcohol in the context of SARS-CoV-2 infection, including the unfounded hypothesis that strong alcohol can kill the virus. Numerous articles perpetuated the notion that light alcohol such as beer or wine could stimulate the immune system to combat the coronavirus [7,35,36]. This misinformation had severe consequences, as evidenced by the 60 people who lost their sight and nearly 6000 individuals hospitalized after consuming methanol as a “preventive” measure [7,36]. While a study from Hong Kong suggested that around 19% of respondents encountered content suggesting a beneficial link between alcohol consumption and the prevention of SARS-CoV-2 infection [37], there is a scarcity of scientific reports estimating the overall prevalence of fake news on this subject. It is worth noting that for the topic of dietary interventions, the term “misinformation” is used instead of “disinformation.” This is because some of the recommendations might have been well-intentioned but were ineffective or misguided self-help strategies. Hence, such information does not fall into the category of intentionally misleading (disinformation).

In summary, while there are numerous reports highlighting the harmful effects of medical disinformation and misinformation on public health, there remains a dearth of studies focusing on the dynamics of the dissemination of unreliable online health information.

## Methods

### Preliminary Questionnaires

The initial phase of data collection involved identifying areas where topics were likely to generate unreliable content. We began by pinpointing myths and false beliefs commonly propagated by patients in doctors’ offices and hospital wards, recognizing that these beliefs realistically impede doctors from effectively treating patients. To facilitate identification, a questionnaire was developed and administered for this purpose (Multimedia Appendix 1). The questionnaires were gathered at the Clinical Hospital of the Medical University of Bialystok in Poland and through an online survey. The online survey was made possible through collaborations with institutions that have signed letters of intent with the Medical University of Bialystok, including the College of Family Physicians and the Polish Society of Cardiology. Data collection spanned from November 29, 2021, to December 26, 2021 (refer to Multimedia Appendix 1). The questionnaire encompassed inquiries about the physician’s age, gender, specialization, length of service in the profession, and place of work. Additionally, an open-ended question was included: “What false medical information do you encounter in your professional practice when talking to patients?,” with a total of 121 physicians responding. The average age of the doctors was 41.1 (SD 11.4) years, with women constituting 64.5% (78/121). The average duration of employment in the profession was 15.2 (SD 11.4) years. The physicians had various specialties, including internal medicine (n=41), cardiology (n=34), pediatrics (n=21), family medicine (n=18), surgery (n=8), hematology (n=6), allergology (n=6), ophthalmology (n=4), rheumatology (n=1), pulmonology (n=1), emergency medicine (n=1), and doctors in training (n=3). Some physicians held multiple medical specialties. In terms of workplace distribution, the majority of doctors worked exclusively in hospitals (n=70), while others worked in a combination of hospitals, public, and private clinics (n=8); hospitals and public clinics (n=8); or hospitals and private clinics (n=19). Additionally, some respondents were exclusively employed in public clinics (n=11) or private clinics (n=5). The physicians provided descriptions of all the topics they had encountered in their professional practice and quoted specific questions posed by patients. Through the analysis of the questionnaires, we identified common problems and subsequently categorized them into 9 groups (topics). These topics were delineated based on their consistent scope and association with false medical information.

### Web Page and Social Media Engagements Retrieval Using BuzzSumo

Subsequently, queries were formulated based on the identified topics (problem groups). These topics were then assigned to each member of another team of physicians, who conducted searches using the DuckDuckGo search engine. The queries comprised single words or several words forming a specific meaningful phrase. Physicians considered only queries that resulted in unreliable websites. In total, 274 queries were collected through this method.

**Figure 1:**
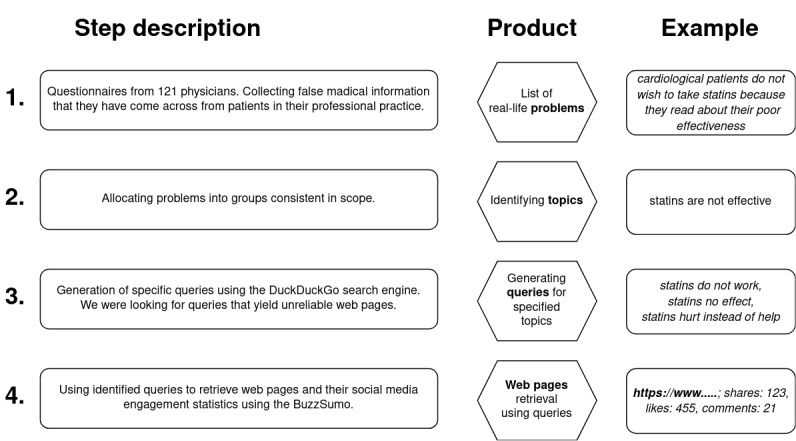
Four subsequent stages of web search research. The figure includes a step description, the product of the step, and an example.

Subsequently, the BuzzSumo Enterprise Application (BuzzSumo Limited) was used [38]. BuzzSumo, a social media analytics tool, searches the internet for web documents based on queries and generates engagement reports, encompassing comments, likes, and shares on social media. This tool collects data from various platforms, including Facebook, Pinterest, Reddit, and Twitter, to compile a list of article web pages with the highest online engagement. *Engagement,* in this context, is defined as the cumulative number of interactions users have with a specific article link, encompassing actions such as “liking,” “sharing,” and “commenting.” Upon entering a query, the application provides statistics for the linked pages. The subsequent steps of the web search process are illustrated in Figure 1.

The search was restricted to 2 specific periods: (1) January 1, 2021, to December 31, 2021; (2) January 1, 2022, to December 31, 2022. Searches were filtered by time and language, specifically Polish. The analysis occurred in 2 consecutive phases. The first phase covered the entirety of 2021 and took place in January 2022. The second phase occurred in January 2023 and encompassed the entirety of 2022. This approach allowed us to evaluate current websites, recognizing that the online environment is dynamic, and web pages are frequently deactivated or cease to generate traffic. This observation is further supported by our findings. Even though identical queries were used to search websites during both periods, there was no repetition of websites selected in 2021 during the search in 2022. This lack of overlap underscores the dynamic nature of the online environment, where web pages frequently change or cease to be relevant over time.

**Figure 2:**
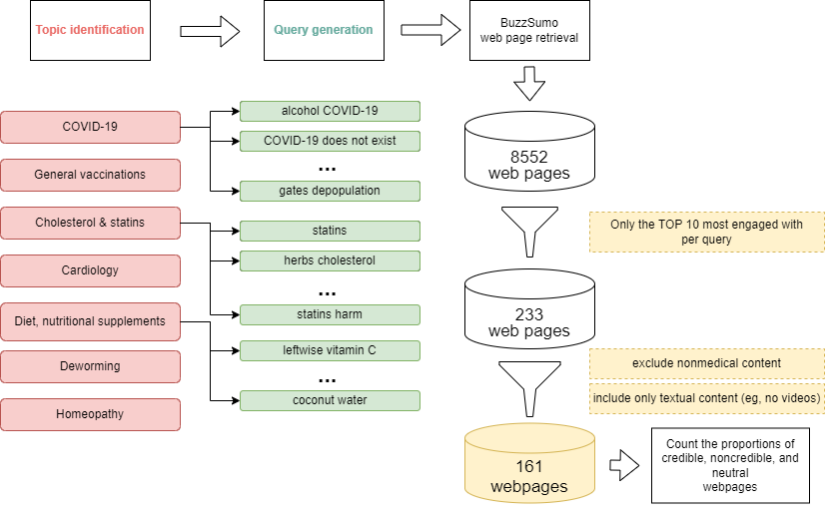
The figure contains a detailed description of the subsequent stages of the web search research, with the year 2021 as an example. Topic identification, query generation, web page retrieval, and the subsequent web page filtration are depicted.

For inclusion in the analysis, only pages that garnered at least ten social media engagements and contained text were considered; videos were not evaluated. Subsequently, the 10 most popular pages per query were analyzed, with “popular” referring to those with the highest total engagements. Each web page underwent review by an experienced doctor, holding at least five years of professional experience, who is also a scientist at the Medical University of Bialystok. To facilitate clarity in assessing the web pages, categories were introduced based on their content: medical, nonmedical, and difficult to evaluate. Of the 274 queries, only 58 yielded medical content in 2021, and 32 in 2022, as illustrated in Figure 2.

Regarding web pages categorized as medical, the content was evaluated in terms of credibility, classified as credible, noncredible, or neutral. Specialists in the pertinent medical field assessed websites relevant to their expertise, such as cardiologists for topics on statins and infectious disease specialists for COVID-19 topics. The assessment of health information credibility in the news involved comparing it with current textbooks, guidelines, and scientific articles in the respective field. The evaluation adhered to evidence-based medicine standards, and the criteria used for assessment are detailed in Table 1.

**Table 1:** Content categories.

Category	Content
Credible medical websites	Contains verifiable/up-to-date medical information and does not contain information intended to mislead the viewer/reader.
Noncredible medical websites	Contains false medical information. Contains unverified medical information. Contains outdated medical information that is inconsistent with current standards. Contains medical information consistent with evidence-based medicine, but the implications of the fact(s) presented are falsely exaggerated. Contains partially not credible medical information: miscalculations or erroneous conclusions from true analyses; a mixture of credible and not credible information. Contains noncredible medical information or information inconsistent with evidence-based medicine, but part of the text softens the implication of the sentence.
Neutral medical websites	Contains information related to medicine, but describes a story related to a disease, reports on a medical encounter, and presents regulations in the health care sphere.
Nonmedical website	Does not contain information related to medicine.

The data were collected and analyzed using Microsoft Excel (Microsoft, Inc.). Descriptive statistics were computed for categorical variables, presenting the frequencies of the variables under investigation in this scientific study. The chi-square test was used to assess the distribution of medical web pages categorized as credible, noncredible, or neutral between the years 2021 and 2022. The chi-square test was used to ascertain whether there was a statistically significant (*P*<.05) difference in the distribution of both the number and popularity of these web pages across the mentioned categories. The aim was to investigate whether there were any shifts in the credibility classification of web pages and their corresponding popularity over the 2 years. In cases where expected values were less than 5, the Fisher exact test was used. Considering the least represented data, the popularity of neutral medical web pages for 2021 and 2022 was excluded from the analysis due to computational constraints associated with the Fisher exact test.

### Ethical Considerations

The study used only anonymized or de-identified data. Data was collected using BuzzSumo, which uses aggregated, anonymous statistics and does not access personal data. The Bioethics Committee of the Medical University of Bialystok deemed that this study does not meet the definition of a medical experiment, as it is not carried out on human beings or biological material taken from a human being for scientific purposes. Therefore, the approval of the Bioethics Committee was not required.

## Results

### Preliminary Questionnaires

The most common topics indicated by doctors were COVID-19 infection, COVID-19 vaccination, vaccination against other infectious diseases, hypercholesterolemia and statins, hypertension, nutritional supplements, and antibiotics. A list of topics is shown in Table 2.

**Table 2:** The list of topics (problem groups) indicated by doctors in questionnaires, the number of times the topic was mentioned, and the resulting number of queries generated from this topic for data collection purposes.

Number	Topics	Times the topic was indicated by doctors, n	Queries, n
1	Coronavirus disease 2019 (COVID-19)	74	85
2	General vaccinations	64	18
3	Cholesterol and statins	52	45
4	Cardiology	27	68
5	Diet, nutritional supplements, and alcohol	26	32
6	Antibiotics	12	12
7	Deworming	4	9
8	Lyme disease	2	1
9	Homeopathy	1	4

### Web Page and Social Media Engagements Retrieval Using BuzzSumo

In total, 8552 web pages were collected in 2021, decreasing to 6404 in 2022. COVID-19 topics held the top spot in popularity during 2021, whereas cholesterol and statins took the lead in 2022. Detailed topic-specific data are provided in Table 3.

After following the steps outlined in Figure 1, a total of 161 web pages from 2021 and 105 from 2022 underwent analysis. The filtering process is illustrated in Table 4.

**Table 3:** The number of all web pages based on the queries, retrieved by BuzzSumo (2021-2022).

Topics	All web pages retrieved by BuzzSumo in 2021, n	All web pages retrieved by BuzzSumo in 2022, n
Coronavirus disease 2019 (COVID-19)	5448	1495
General vaccinations	468	59
Cholesterol and statins	1152	3282
Cardiology	1326	1456
Diet, nutritional supplements, and alcohol	150	23
Deworming	3	8
Homeopathy	8	80
Total	8552	6404

**Table 4:** Subsequent stages of web search research according to Figure 1.

Stages	Web pages in 2021, n	Web pages in 2023, n
Web pages retrieved by BuzzSumo using selected queries	8552	6404
Web pages that provoked engagements at least ten times (videos were not evaluated)	233	137
Web pages considered by experts as containing medical content	161	105

**Figure 3:**
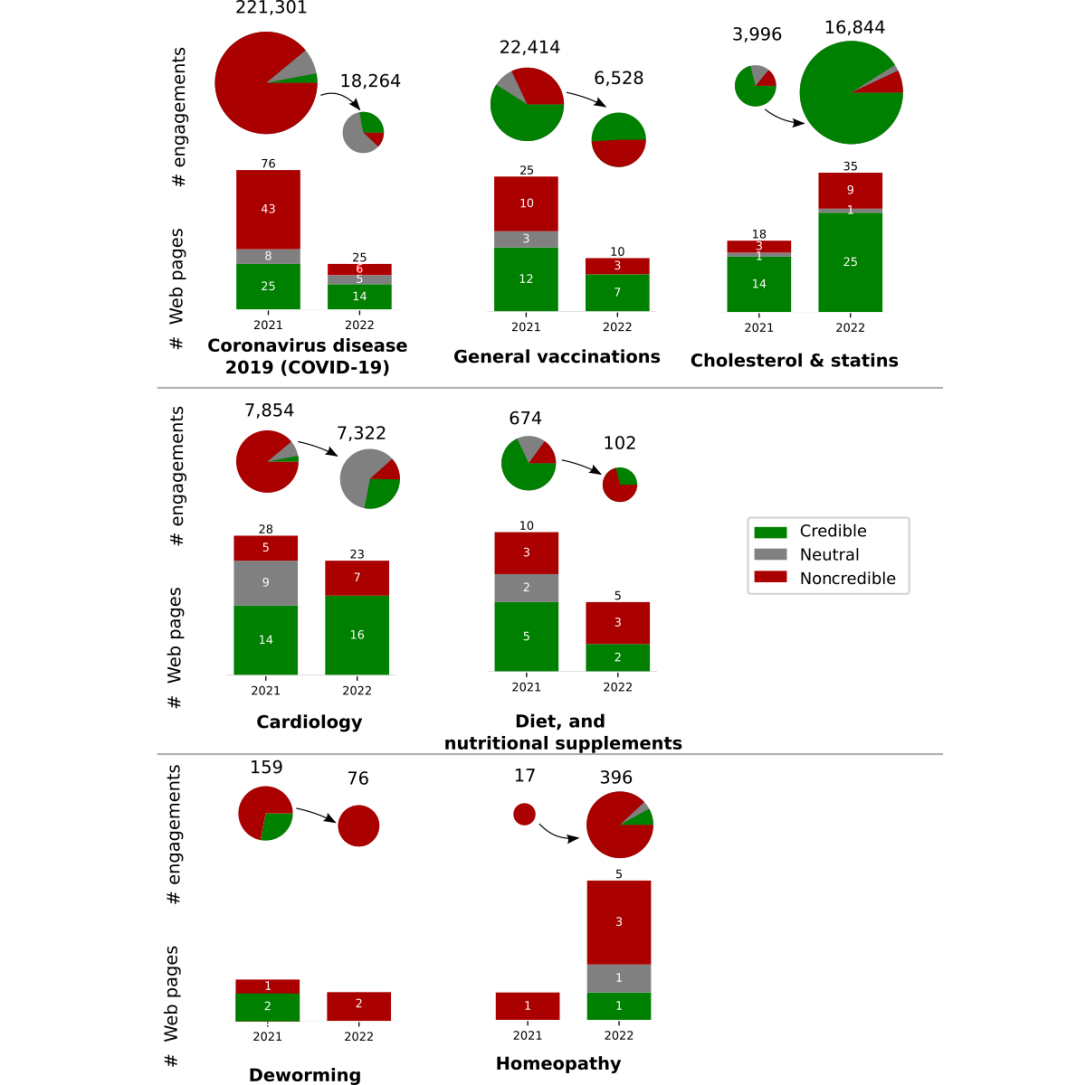
The figure shows the differences in the prevalence and interactions on social media of non-credible, credible, and neutral web pages in the two periods: (1) January 1, 2021 to December 31, 2021; (2) January 1, 2022 - December 31, 2022. The bars indicate the number of websites that exceeded the social media engagement threshold (top 10 web pages mostly interacted with per search query) in a given period related to a given medical topic. Pie charts show the number of interactions (shares, likes, comments) with non-credible, credible, and neutral web pages over a given period, on a given medical topic. The aggregated social media engagement data was retrieved using the BuzzSumo Enterprise Application.

In 2021, web pages related to the COVID-19 topic were the most popular, accumulating 221,301 engagements, followed by the general vaccinations topic, which scored 22,414 engagements. Similarly, in 2022, the most popular web pages were those associated with the COVID-19 topic, generating 18,264 engagements, followed by cholesterol and statins, which garnered 16,844 engagements. The topics most tainted with fake or noncredible content in 2021 were COVID-19 (43/76, 57%), followed by general immunization (10/25, 40%). In 2022, diet, nutritional supplements, and alcohol took the lead in content pollution (3/5, 60%), followed by general vaccinations (3/10, 30%). The percentage of fake or noncredible web pages related to COVID-19 and general vaccinations decreased from 57% (43/76) to 25% (6/24) and 42% (10/25) to 30% (3/10), respectively. Conversely, the percentage of fake or noncredible web pages related to cholesterol/statins and cardiology increased from 11% (3/28) to 26% (9/35) and 13% (5/38) to 26% (6/23), respectively (Figure 3).

In Table 5, the count of medical web pages, as assessed by medical experts, is categorized into credible, noncredible, or neutral. Web pages labeled as medical neutral did not contain diagnostic or treatment-related medical information but mainly focused on health stories or described changes in medical regulations or conferences. In 2021, the count of medical web pages rated as credible, noncredible, and neutral was 72, 66, and 23, respectively. In 2022, these figures changed to 66, 32, and 7, respectively. Notably, the percentage of noncredible content from all web pages in 2022 was much lower at 30.5% (32/105), compared with 41% (66/161) in 2021.

In 2021, credible web pages garnered 24,398 engagements, increasing to 30,301 engagements in 2022. Web pages featuring fake news generated 210,018 engagements in 2021 and 7942 engagements in 2022. Further details are presented in Table 6.

**Table 5:** The number of medical web pages, divided into credible, noncredible, and neutral (2021-2022).

Topics	2021				2022				*P* value
	Credible medical web pages, n	Noncredible medical web pages, n	Neutral medical web pages, n	Credible medical web pages, n		Noncredible medical web pages, n	Neutral medical web pages, n		
Coronavirus disease 2019 (COVID-19)	25	43	8	14		6	5	.02^a^	
General vaccinations	12	10	3	7		3	0	.47^b^	
Cholesterol and statins	14	3	1	25		9	1	.76^b^	
Cardiology	14	5	9	17		6	0	.007^b^	
Diet, nutritional supplements, and alcohol	5	3	2	2		3	0	.62^b^	
Deworming	2	1	0	0		2	0	.40^b^	
Homeopathy	0	1	0	1		3	1	>.99^b^	
Total	72	66	23	66		32	7	.01^a^	

^a^Chi-square test.

^b^Fisher exact test.

**Table 6:** The number of aggregated engagements of medical web pages, divided into credible, fake news, and neutral (2021-2022).

Topics	2021, n				2022, n				*P* value
	Credible medical web page popularity, n	Noncredible medical web page popularity, n	Neutral medical web page popularity, n	Credible medical web page popularity, n		Noncredible medical web page popularity, n	Neutral medical web page popularity, n		
Coronavirus disease 2019 (COVID-19)	6739	197,100	17,462	5138		2145	10,981	<.001^a^	
General vaccinations	13,234	7226	1954	3325		3203	0	<.001^b^	
Cholesterol and statins	2831	579	586	15,324		1228	292	<.001^a^	
Cardiology	1090	4883	1881	6454		868	0	<.001^b^	
Diet, nutritional supplements, and alcohol	459	99	116	30		72	0	<.001^b^	
Deworming	45	114	0	0		76	0	<.001^b^	
Homeopathy	0	17	0	30		350	16	.33^b^	
Total	24,398	210,018	21,999	30,301		7942	11,289	<.001^a^	

^a^Chi-square test.

^b^Fisher exact test.

## Discussion

### Principal Findings

Numerous researchers have endeavored to develop algorithms for identifying disinformation and misinformation on the web. Machine learning protocols and artificial intelligence have been used to detect fake news related to SARS-CoV-2 infection on social media [13,14,39]. Iwendi et al [14] introduced new features to accurately recognize COVID-19–related fake news in online information, achieving an accuracy rate of over 86%. The implementation of machine learning–supported measures during the COVID-19 pandemic to curb the dissemination of fake news appears to have proven effective. Nevertheless, it is imperative to implement such interventions continuously, particularly in emerging medical fields vulnerable to generating unreliable content, given the significant impact on the community.

This study primarily addressed the perspective of the layperson, recognizing the challenges they face in comprehending medical information and identifying reliable sources. Laypersons are consequently at risk of coming across noncredible information [9,25]. By contrast, health care providers rely on established, reliable sources of medical knowledge grounded in evidence-based medicine [10].

In the last 2 years, COVID-19 emerged as the most commonly reported topic generating “fake news” in doctors’ daily clinical practice. Despite the availability of evidence-based medicine guidelines for the treatment of COVID-19 [40], our study revealed that in Poland, the most popular web pages still centered around the use of alternative methods to treat SARS-CoV-2 infection, such as amantadine. Notably, the proportion of noncredible web pages in the selected sample regarding COVID-19 was 0.57 in 2021, which reduced to 0.24 in 2022. It is noteworthy that credible web pages were less prevalent on the web, with a considerably lower number of engagements compared with noncredible ones in both years (6739 vs 221,301 and 5138 vs 18,264). This suggests that false information about COVID-19 remained prominent on the web in 2021. However, the significant decrease in total engagements related to noncredible content may indicate that interventions to detect, remove, and correct disinformation about COVID-19, implemented by major tech companies such as Google and Facebook, had a positive impact on curtailing the spread of fake news. On-platform interventions comprised highlighting, surfacing, and prioritizing content from authoritative sources, as well as cooperating with fact-checkers and health authorities to flag and remove disinformation [41]. Another conceivable factor contributing to the diminished spread of COVID-19–related fake news could be a waning interest in the pandemic. Possible reasons are people growing accustomed to it, the easing of restrictions, and the emergence of another global crisis in 2022, namely, the war in Ukraine. Nevertheless, our study has identified a decrease in interest in noncredible information about COVID-19 from 2021 to 2022. Interestingly, the popularity of other topics, particularly cholesterol/statins and cardiology, has seen an increase. It appears that new topics are effectively “filling up the vacuum” created by the declining interest in the virus.

In the case of the topic “cholesterol and statins,” social media witnessed a substantial surge in interest in this field between 2021 and 2022. The number of aggregated total engagements grew more than fourfold, escalating from around 4000 to approximately 16,800. This surge coincided with an increased interest in other cardiology topics and a decline in interest in COVID-19. While most web page contents were evaluated as credible, noncredible content accounted for 11% (3/28) in 2021, rising to 26% (9/35) in 2022. Notably, during the period of heightened interest in the topic of “cholesterol and statins,” the percentage of content classified as noncredible also experienced an increase. This phenomenon was corroborated in the other studied categories. Hypercholesterolemia is a condition that does not manifest clinical symptoms directly. It remains asymptomatic for an extended period, impacting life comfort only when complications such as stroke or myocardial infarction arise. Patients often visit doctor’s offices for unrelated reasons, only to receive a diagnosis of hypercholesterolemia. Subsequently, doctors assess the need for pharmacological treatment. As a consequence of this unique scenario, doctors’ competence in this field is frequently called into question. Given the general low trust in the medical profession in Poland, coupled with heightened fears of side effects and distant prospects of clinical benefits, patients often seek alternative information on the internet and social media. The increased interest in the subject serves as fertile ground for the creation of fake news content.

Based on the results obtained, the topic of deworming generated the least engagement. However, this issue was identified as prevalent among patients in our initial questionnaire. Surprisingly, the number of engagements for noncredible web pages concerning this topic surged from 71.7% (114/159) in 2021 to a staggering 100% (76/76) in 2022. While the absolute numbers of web pages are modest (3 and 2 for 2021 and 2022, respectively), the absence of any credible source of information about this topic in 2022 should raise alarms within the medical community. Qualitative insights uncovered the influential role of social media influencers in shaping the narratives surrounding deworming. These findings suggest that deworming topics might not generate a substantial number of clicks, but they carry a potent and convincing message for the layperson. A study reveals that the analysis of internet searches accurately predicts seasonal peaks in hairworm numbers, identifying host characteristics that align precisely with scientific data [42]. Drawing from this study, we can infer that lay internet users frequently search for information about parasites but seldom land on reliable sources.

Throughout the pandemic, we all encountered the phenomenon known as an “infodemic,” an influx of an extensive amount of information, including false and fake news, during the outbreak of a particular disease [15]. While COVID-19 serves as a vivid illustration of how harmful and contagious medical disinformation can be, there remain other health topics where online disinformation is rampant. These topics were already sources of disinformation before the COVID-19 pandemic and continue to pose a significant threat to society. One of the strengths of our study is that the analysis of most unreliable topics is grounded in clinical practice. Additionally, the evaluation of the websites was conducted by an experienced team of clinicians.

### Limitations

The initial questionnaire had a limited sample size with only 121 respondents. Unfortunately, during the COVID-19 pandemic, doctors were facing overwhelming workloads. Nevertheless, the respondents represented a diverse group of doctors from various specialties, working in different settings—both inpatient and outpatient—and spanning different age groups. Notably, these doctors provided comprehensive descriptions of all the problems they had encountered with disinformation in their professional work. The questionnaire did not prescribe specific topics for discussion. Doctors, based on their clinical experience, independently described these phenomena. Despite the relatively small number of respondents, this approach ensured a comprehensive exploration of the topics.

Examining only the first 10 websites selected with BuzzSumo using the queries might not provide a complete picture of the spread of medical fake news across the web. Nevertheless, given our limited resources, primarily the time of the experts, we endeavored to capture the dynamics of the most popular content as accurately as possible. Another limitation is the selection of websites in both time intervals using precisely the same queries. In the second time slot, there is a possibility that new medical topics were not included in the selection. To address this limitation, we plan to reintroduce the questionnaire and assess the dynamics to identify potential new and emerging topics generating significant medical noncredible content on the internet.

### Conclusions

Our analysis underscores several critical areas for improvement in interventions aimed at halting the spread of noncredible online health information. These can be summarized as follows:

- While interventions implemented by big tech companies have proven effective in combating COVID-19 disinformation, the surge in the dissemination of noncredible content between 2021 and 2022 in other topics underscores the need for these interventions to extend beyond COVID-19–related issues.
- Beyond COVID-19, the topics of “cholesterol and statins” and “cardiology” stand out as significant generators of unreliable content. Initiating interventions with a focus on these topics may be a worthwhile consideration.
- The “deworming” topic illustrates that online activity does not consistently mirror the actual spread of disinformation. Despite being frequently indicated in questionnaires, this topic generated only a few web pages, with minimal total engagements. It is noteworthy that all web pages found in 2022 related to this topic were noncredible. This underscores the significance of periodically repeating questionnaires among physicians.
- The “deworming” case also brings to light the inadequacy of coverage for certain medical topics by credible sources of online health information. There should be a special emphasis on addressing such topics.
- Continuous monitoring of the dynamics in the dissemination of disinformation for topics that are frequently shared, commented on, or liked is essential. This proactive approach allows for timely reactions to potential future infodemics.

## Appendix

Multimedia Appendix 1 The preliminary questionnaire body.

## Abbreviations

MMR: measles, mumps, and rubella
WWW: World Wide Web
