# Supplementary figures and images for: Medical Misinformation in Polish on the World Wide Web During the COVID-19 Pandemic Period: Infodemiology Study
Source: J Med Internet Res. 2024 Mar 29;26:e48130. doi: 10.2196/48130 (PMC10984342; doi:10.2196/48130)

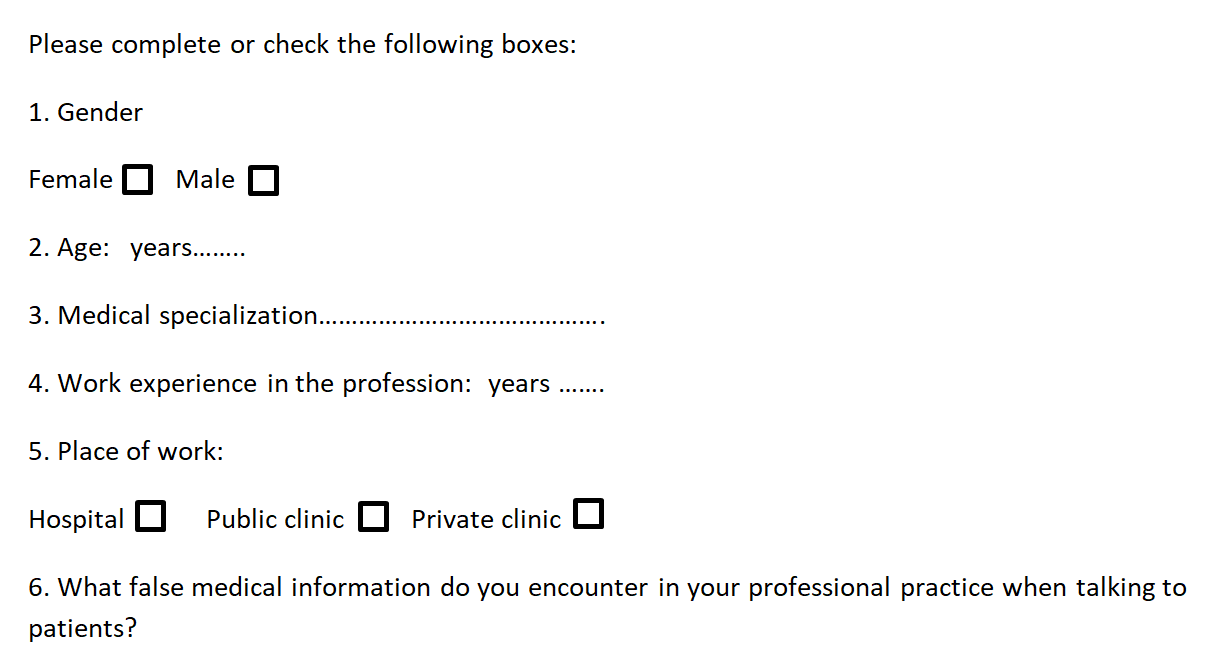

Supplement: Multimedia Appendix 1 [file jmir_v26i1e48130_app1.png]
